# Metabolic Responses to Carbohydrate Ingestion during Exercise: Associations between Carbohydrate Dose and Endurance Performance

**DOI:** 10.3390/nu10010037

**Published:** 2018-01-03

**Authors:** Michael L. Newell, Gareth A. Wallis, Angus M. Hunter, Kevin D. Tipton, Stuart D. R. Galloway

**Affiliations:** 1 Department of Life Sciences, Faculty of Science and Technology University of Westminster, London W1W 6UW, UK; 2 School of Sport, Exercise and Rehabilitation Sciences, University of Birmingham, Birmingham B15 2TT, UK; g.a.wallis@bham.ac.uk; 3 Physiology, Exercise and Nutrition Research Group, Faculty of Health Sciences and Sport, University of Stirling, Stirling FK9 4LA, UK; a.m.hunter1@stir.ac.uk (A.M.H.); k.d.tipton@stir.ac.uk (K.D.T.); s.d.r.galloway@stir.ac.uk (S.D.R.G.)

**Keywords:** glucose, fat oxidation, exogenous, fatty acids, hepatic glucose output

## Abstract

Carbohydrate (CHO) ingestion during exercise lasting less than three hours improves endurance exercise performance but there is still debate about the optimal dose. We utilised stable isotopes and blood metabolite profiles to further examine metabolic responses to CHO (glucose only) ingestion in the 20–64 g·h^−1^ range, and to determine the association with performance outcome. In a double-blind, randomized cross-over design, male cyclists (*n* = 20, mean ± SD, age 34 ± 10 years, mass 75.8 ± 9 kg, peak power output 394 ± 36 W, VO_2max_ 62 ± 9 mL·kg^−1^·min^−1^) completed four main experimental trials. Each trial involved a two-hour constant load ride (185 ± 25 W) followed by a time trial, where one of three CHO beverages, or a control (water), were administered every 15 min, providing 0, 20, 39 or 64 g CHO·h^−1^. Dual glucose tracer techniques, indirect calorimetry and blood analyses were used to determine glucose kinetics, exogenous CHO oxidation (EXO), endogenous CHO and fat oxidation; and metabolite responses. Regression analysis revealed that total exogenous CHO oxidised in the second hour of exercise, and suppression of serum NEFA concentration provided the best prediction model of performance outcome. However, the model could only explain ~19% of the variance in performance outcome. The present data demonstrate that consuming ~40 g·h^−1^ of CHO appears to be the minimum ingestion rate required to induce metabolic effects that are sufficient to impact upon performance outcome. These data highlight a lack of performance benefit and few changes in metabolic outcomes beyond an ingestion rate of 39 g·h^−1^. Further work is required to explore dose-response effects of CHO feeding and associations between multiple metabolic parameters and subsequent performance outcome.

## 1. Introduction

During prolonged steady state exercise, endogenous glycogen stores and circulating plasma glucose are key substrates for energy provision. Fatigue is often reported to coincide with the depletion of endogenous carbohydrate (CHO) stores and the dysregulation of circulating plasma glucose concentration [1,2,3]. Ingesting CHO improves performance and extends exercise duration via a range of proposed mechanisms including: better maintenance of circulating plasma glucose [1], higher rates of exogenous [4] and total CHO oxidation, and endogenous glycogen sparing [5]. These proposed mechanisms do not occur in isolation but occur together facilitating force production and improving performance and exercise capacity.

Early research by Coyle et al. [1] reported that feeding CHO maintained blood glucose concentration and CHO oxidation rates, and in turn, exercise capacity increased 33% (3.02 versus 4.02 h) significantly in comparison to a water control. In a follow-up study [2] participants exercised to exhaustion and were then provided with either no CHO, ingested CHO, or infused CHO. Both CHO provision conditions increased exercise duration on commencement of exercise in comparison to no CHO. However, only the infusion condition maintained blood glucose concentration sufficiently to subsequently extend exercise duration above that of the CHO ingestion trial. The authors concluded that the maintenance of blood glucose concentration was the critical factor for maintaining sufficient CHO oxidation rates to extend exercise capacity.

Further research has indicated that the maintenance of higher CHO oxidation can be primarily explained by an increase in exogenous CHO oxidation rates [4]. An elevation in exogenous CHO oxidation rate and enhanced endurance exercise performance are now believed to be directly associated, despite little systematic evaluation to date. As a result, increasing exogenous CHO oxidation rate is thought to be essential for the enhancement of endurance performance when ingesting CHO throughout a range of 20–100 g·h^−1^. This relationship has led some researchers to hypothesise that maximising exogenous CHO oxidation rate, through use of glucose: fructose combinations at high feeding rates will result in further performance gains [6]. However, most studies examining performance benefits of multiple transportable CHO ingestion have been conducted in comparison to isocaloric single source CHO. The findings using this model are likely to be confounded by gastrointestinal issues when ingesting single source CHO at high feeding rates. At lower feeding rates, Smith et al. [4] demonstrated that the largest improvement in performance occurred when ingesting 60 g·h^−1^ in comparison to 15 or 30 g·h^−1^. The 60 g·h^−1^ ingestion rate also resulted in the highest exogenous CHO oxidation rate. These authors reported a non-significant but ‘likely’ 2.3% improvement in performance when comparing 60 vs. 30 g·h^−1^ suggesting a dose-response effect of CHO feeding rate. We recently reported that a 2.3% performance gain would not necessarily be ‘likely’ due to typical variance observed in performance outcome measures [7] when using a more suitably powered design. In addition, there has yet to be an extensive exploration of the association between multiple metabolic variables and subsequent exercise performance outcomes using a dose-response investigation. Thus, more work remains to be done to determine the key factors driving performance improvement in exercise lasting <3 h. In our previous work [7] a lack of any further improvement in performance when feeding 64 g·h^−1^ in comparison to 39 g·h^−1^ suggests that the metabolic alterations with feeding rates as low as 39 g·h^−1^ could be sufficient to maximise performance gains within this feeding rate range. As such, peak exogenous carbohydrate oxidation rate may not be the sole, or key, determining factor for performance enhancement during exercise lasting less than 3 h with the single source CHO doses studied.

Feeding CHO during exercise influences the usage of endogenous glycogen stores. Several studies have assessed endogenous glycogen utilisation using stable isotopes during 1–2 h of moderate intensity exercise. McConnell et al. [8] provided participants with 100 g·h^−1^ of CHO during 2 h of exercise at 69 ± 1% VO_2peak_. Hepatic glucose output was suppressed in comparison to a control trial and remained close to baseline rates throughout the exercise bout. The authors calculated that a 51% reduction in hepatic glucose production occurred as a result of consuming 100 g·h^−1^ CHO in comparison to the control. Furthermore, Jeukendrup et al. [5] provided 30 and 180 g·h^−1^ of a glucose based CHO beverage during a 2 h moderate intensity exercise bout. They reported reduced fat oxidation rates, increased rate of appearance (Ra) and rate of disappearance (Rd) of glucose, and an increase in the oxidation of exogenous CHO particularly with the higher glucose dose. Endogenous muscle glycogen oxidation rates were not altered with either 30 or 180 g·h^−1^ of CHO in their study. However, liver glycogen breakdown was reduced when consuming 30 g·h^−1^, and completely inhibited when consuming 180 g·h^−1^ of CHO. These observations suggest that only when very high doses of glucose are ingested can hepatic glucose production be completely inhibited. Smith et al. [4] estimated a stepped reduction in the contribution of liver glycogen to total CHO oxidation during the second hour of their submaximal exercise bout whilst consuming 15, 30 and 60 g·h^−1^ of CHO. Interestingly, all three studies indicate that muscle glycogen was not spared with any ingestion rate provided. These data suggest that a focus on hepatic glycogen sparing is required when considering factors likely to influence subsequent performance outcomes.

The amount of CHO to ingest for optimal endurance performance has been widely debated. A consensus has been reached that the maximal exogenous CHO oxidation rate that can be achieved with glucose (single source CHO) ingestion is around ~1 g·min^−1^. As previously mentioned, Smith et al. [4] suggested the existence of a dose response relationship between CHO ingestion rate and endurance exercise performance enhancement when feeding 0, 15, 30 and 60 g·h^−1^ of glucose. However, their initial study was underpowered. Their study was followed up with a multicentre investigation which presented evidence for a curvilinear dose response relationship with ingestion rates of a multi-source CHO beverage spanning 0 to 120 g·h^−1^ with a statistically optimal ingestion rate reported as 78 g·h^−1^. However, whether maximal exogenous oxidation rates driven by higher CHO ingestion rates result in optimal performances during endurance tasks requires further metabolic analysis. Until now our previously published work is the most suitably powered and most statistically robust study design to indicate the lack of a clear dose response relationship with ingestion rates between 20 and 64 g·h^−1^ [7]. However, from these data alone we are unable to determine what the underlying physiological explanations were for the plateau in performance. We now present the metabolic data to explore these performance changes more comprehensively.

As such, in the present manuscript we aimed to explore the metabolic responses to submaximal endurance exercise with CHO ingestion rates between 0 and 64 g·h^−1^. We specifically aimed to: examine glucose kinetics and quantify or estimate the total substrate usage from exogenous and endogenous glycogen stores by utilising stable isotopic tracers; measure key circulating metabolites; quantify the percentage contribution of key substrates throughout the exercise bout. We hypothesised that during exercise lasting <3 h there would be a minimum effective dose of CHO required to result in optimal metabolic responses, and endogenous CHO sparing, linked to improved performance outcome. We also hypothesized that both exogenous CHO oxidation rate and reduction in hepatic glucose production would be the key parameters most closely associated with performance outcomes.

## 2. Materials and Methods

### 2.1. Participants

Twenty trained male cyclists were recruited from regional cycling and triathlon clubs. The mean (±SD) characteristics of the participants were: age 34.0 (±10.2) years, body mass 74.6 (±7.9) kg, stature 178.3 (±8.0) cm, peak power output (PPO) 393 (±36) W, power output (PO) at lactate threshold 206 (±30) W and VO_2max_ 62 (±9) mL·kg^−1^·min^−1^. Participants were required to have been training for >6 h/week for >3 years. Each individual had the procedures and associated risks explained prior to providing written informed consent to participate in the study. The study was approved by the University of Stirling, Research Ethics Committee (SSREC number 604) in accordance with the Declaration of Helsinki. In some circumstances, not all participants were included in all datasets. Unfortunately, 2 participants had to be removed from all stable isotope and substrate use data due to measurement errors. Hence, the characteristics of participants included in the stable isotope analyses were: body mass 76.9 (±8.4) kg, stature 178.7 (±8.1) cm, PPO 392 (±34) W, VO_2max_ 61.2 (±8.2) mL·kg^−1^·min^−1^ and PO at lactate threshold 206 (±30) W.

### 2.2. Pretesting

Following pre-screening, on week one of six, after a ten hour overnight fast, participants performed a two-part incremental cycle test (Lode Excalibur Sport, The Netherlands) to determine lactate threshold (LT), maximal oxygen uptake (VO_2max_), and peak power output as described previously [7]. The mean ± SD lactate concentration at LT was 2.1 ± 0.4 mmol·L^−1^ corresponding to an intensity of 52 ± 6% of PPO for LT which is typical of other studies utilising a similar protocol [9]. The test end time and power output of the final stage was used to calculate peak power output (PPO) using the following Equation (1) [10]:

PPO = W_final_ + ([*t*/60] × PI) (1)

where, W_final_ = the power output of the final completed stage in (watts), *t* = the time spent in the final uncompleted stage (seconds), 60 = the duration of each stage (seconds) and PI = the increase in power output between each stage (W). Maximal oxygen uptake (VO_2max_) was assessed via an automated online gas analyser (Oxycon Pro, Jaeger, Wuerzerberg, Germany). VO_2max_ was determined as the highest average VO_2_ captured over a 30 s period.

### 2.3. Design

In a double blind, placebo controlled, randomised cross-over study design participants visited the laboratory for 5 experimental trials (1 preliminary and 4 intervention) over a five-week period. They completed one visit per week commencing each trial on the same day of the week and at the same time of day. On the first of these trial visits participants completed a full familiarisation. The familiarisation trial and the four subsequent intervention trials consisted of a 120 min steady state submaximal cycle ergometer ride at 95% lactate threshold (185 ± 25 W). Participants were asked to record their habitual dietary intake for 48 h prior to visit one and replicate this dietary intake for the two days prior to each subsequent visit. Additionally, participants were asked to arrive at the laboratory following a ~10 h overnight fast. Water was ingested before and during the familiarisation trial and was consumed at a rate of 1 L·h^−1^. Thereafter, participants consumed in a counterbalanced randomized cross-over design either: 0%, 2%, 3.9% or 6.4% CHO solutions before and during exercise at a fluid ingestion rate of 1 L·h^−1^. The 0% trial was a water control trial. Blood samples, expired gas collection and subjective measures were obtained every 15 min throughout the submaximal ride.

### 2.4. Experimental Trials

On arrival at the laboratory, participants emptied their bladder and bowel prior to nude body mass measurement. Participants then changed into cycling clothing. Teflon catheters were placed into an antecubital vein in each arm. One catheter was attached to a three way stop cock to enable stable isotopic tracer infusion. The second was attached to a 10-cm extension line for multiple venous blood sampling. The sampling line was kept patent with a sterile saline solution flush (2 mL) following each sample collection. A baseline blood sample was drawn (10 mL) prior to commencing the primed (18.54 µmol·kg^−1^) continuous (0.32 µmol·kg^−1^·min^−1^) infusion of 6,6,^2^H_2_ glucose via a calibrated syringe pump (Asena GS Syringe Pump; Alaris Medical Systems, Basingstoke, UK) over 60 min at rest. Further blood samples were drawn at 30 min prior to and at the start of exercise for later determination of isotopic enrichments. The concentration of isotopic tracer in the infusate and the pre and post syringe weights were both determined to confirm the actual infusion rate achieved.

### 2.5. Immediately Pre Exercise

Five minutes prior to the start of exercise, a resting breath sample was collected into an expired gas-sampling bag (Quintron QT00892 GaSampler, Milwaukee, WI 53215, USA). 10 mL gas samples were immediately drawn into a 10 mL syringe from the bag and secured with a three way stop cock. Samples were then extracted with a 21G needle directly, and in duplicate, into evacuated exetainer tubes (Labco, High Wycombe, UK) for the determination of the CO_2_ isotopic ratio of ^13^C/^12^C. Two minutes prior to the start of exercise, a further blood sample was collected and the first bolus of CHO test solution was provided (240 mL). The infusion rate of the deuterated glucose tracer was then doubled at the start of exercise (to 0.64 µmol·kg^−1^·min^−1^) to accommodate for the increased turnover of glucose during exercise and to maintain plasma enrichment.

### 2.6. The 2 h Preload Ride and Performance Task

Participants then completed a 2 h submaximal ride at 95% LT (185 ± 25 W, 59 ± 7% of VO_2max_). In the last 3 min of each 15 min time segment a breath by breath gas capture was obtained for the determination of VO_2_ and VCO_2_ (Oxycon Pro, Mannheim, Germany). Immediately following the expired gas collection participants removed the mouth piece and provided a single end-tidal breath sample into a breath sample bag (Quintron QT00892 GasSampler, Milwaukee, WI 53215, USA) for the determination of ^13^C/^12^C ratio as per the baseline sample. Following the breath sampling a 10 mL blood sample (10 mL) was drawn and stored on ice prior to centrifugation. Finally, participants were asked to rate their perceived exertion [11] before ingesting a volume of test drink (220 mL). This was repeated every 15 min throughout the 2 h ride. Following this a performance task lasting approximately 30 min was conducted and is reported elsewhere [7].

### 2.7. Carbohydrate Solutions

**Figure 1:**
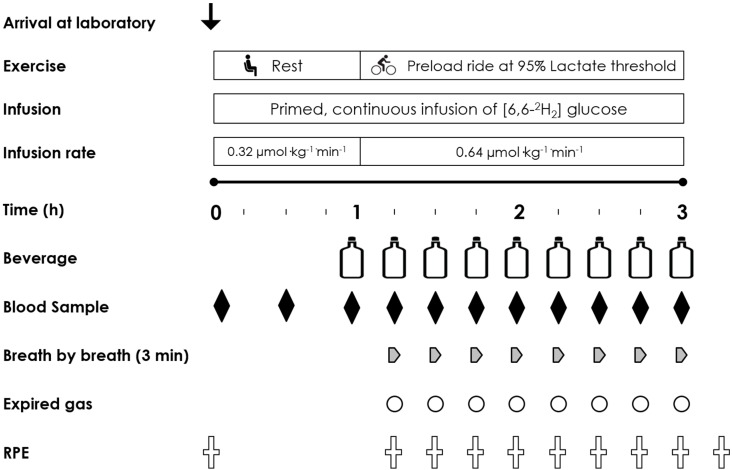
Trial visit time line indicating the infusion rate and time course and the frequency of measures taken throughout each experimental trial visit.

During the 2 h preload ride, each of four solutions were consumed in randomized double-blind fashion: 0% water (control); 2.0%; 3.9%; or 6.4% glucose (single source CHO) based commercially available solutions. All test solutions were maintained at 10 °C and were consumed at a rate of 1 L·h^−1^ providing 0, 20, 39 and 64 g·h^−1^ of CHO respectively. The 20 g·h^−1^ solution contained 37 mg of sodium per 100 mL with the 39 and 64 g·h^−1^ solutions both containing 50 mg per 100 mL. Each solution was initially provided two minutes prior to the start of exercise (240 mL) with subsequent volumes (220 mL) consumed every 15 min. The final drink was provided at 120 min of exercise. All solutions except for the 0% were enriched by adding 50 mg L^−1^ of U-^13^C_6_ glucose (Cambridge Isotopes, Cambridge, UK) during preparation by the laboratory technician. The trial day experimental protocol is shown in Figure 1.

### 2.8. Analyses and Calculations

#### 2.8.1. Blood

Blood samples were collected in EDTA-containing vacutainers and spun in a centrifuge at 3500 rpm for 10 min at 4 °C. Aliquots of plasma were then frozen and stored at −80 °C until further analysis. Plasma glucose, non-esterified fatty acids (NEFA), and lactate were analysed using enzymatic methods on an automated analyser (Ilab Aries, Instrumentation Laboratory, Warrington, UK). Plasma insulin and adrenaline concentrations were analysed using commercially available ELISA kits (Dimedic International, Hamburg, Germany and IBL International, Hamburg, Germany respectively). Both ELISAs were carried out following the manufacturer’s instructions.

Plasma samples were derivatised for the analysis of [^2^H_2_] glucose and [^13^C] glucose content. Briefly, 150 µL of plasma and 150 µL of distilled water with added hydrochloric acid (pH 2) was added to a glass vial and mixed vigorously for 10 s. 3 mL of methanol: chloroform (2.3:1) (500 mL = 348:152) was then added and mixed on a plate shaker (300 rpm) for 3 min. Samples were then centrifuged at 4 °C at 3500 rpm for 15 min. The supernatant was then pipetted into a new glass vial. Here 2 mL of chloroform and 1 mL of distilled water (pH 2) were added and mixed for 15 min on a plate shaker at 300 rpm. Samples were then centrifuged at 4 °C for 15 min at 3500 rpm. The supernatant was then pipetted into a new glass tube. The glass tubes were then transferred to a nitrogen drying rack and incubated at 40 °C for ~2 h until the vials were dry. Once dried 150 µL of butaneboronic acid (10 mg/1 mL pyridine) was added and mixed on a plate shaker for 15 min. Once mixed samples were then incubated at 95 °C for 30 min before 150 µL of acetic anhydride was added and mixed at 300 rpm for 90 min. Samples were then dried under nitrogen gas and incubated at 40 °C until dry. Samples were prepared for the GC-MS and GC-C-IRMS by adding 150 µL of ethylacetate and mixing for 10 min. [6,6,^2^H_2_] enrichment was determined by gas chromatography mass spectroscopy (GCMS) using selected ion monitoring at molecular weights of 297 and 299 ([^12^C] and [^2^H_2_] respectively). Plasma [^13^C] content was assessed using gas chromatography combustion isotope ratio mass spectroscopy (GC-C-IRMS). Plasma ^13^C glucose enrichment was determined using the method of Pickert et al. [12], modified for use with gas chromatography-combustion-IRMS (GC-C-IRMS). The glucose derivative (1 µL) was injected into the GC (split ratio 1:15) and analysed by GC-C-IRMS (GC, Trace GC Ultra; C, GC Combustion III; IRMS, Delta Plus XP; all Thermo Finnigan, Herts, UK).

#### 2.8.2. ^13^C Breath Samples

Breath samples were analysed in duplicate for ^13^C/^12^C ratio by continuous-flow IRMS (GC, Trace GC Ultra; IRMS, Delta Plus XP; both Thermo Finnigan, Herts, UK).

#### 2.8.3. Substrate Oxidation

Expired gas analysis was used to estimate rates of substrate oxidation from VO_2_ and VCO_2_ every 15 min. These breath measures were averaged every 4 breaths and the mean of these were taken from the last 60 s of a 3-min sampling period. Whole body substrate oxidation calculations were based on those proposed by Jeukendrup and Wallis [13]:

CHO oxidation rate (g·min^−1^) = 4.210 VCO_2_ − 2.962 VO_2_ (2)

Fat oxidation rate (g·min^−1^) = 1.695 VO_2_ − 1.701 VCO_2_ (3)

where VCO_2_ and VO_2_ are measured in litres per minute. Once the rate of substrate usage was determined during each 15-min breath by breath capture, the rates calculated in grams per minute were multiplied by 15 and summed from each time point to provide an estimate of the total substrate use during the whole exercise bout. Protein oxidation was considered as negligible.

#### 2.8.4. Tracer Calculations

The isotopic enrichment in the expired breath samples was expressed as mean difference between the ^13^C/^12^C ratio of the sample and a known laboratory reference standard using the following formula to enable calculation of exogenous carbohydrate oxidation:

Exogenous CHO oxidation (g·min^−1^) = VCO_2_ [(R_exp_ − R_ref_)/(R_exo_ − R_ref_)]/k (4)

where VCO_2_ is in litres per minute, R_exp_ is the observed isotopic composition of expired CO_2_, R_ref_ is the isotopic composition of expired CO_2_ with the ingestion of the placebo, R_exo_ is the isotopic composition of exogenous glucose ingested in the drink and k (0.747 L·g^−1^) is the volume of CO_2_ produced by the complete oxidation of glucose.

#### 2.8.5. Percentage Contribution of Substrates (Second Hour of Exercise)

Once the total amount of exogenous carbohydrate oxidation had been determined, this rate was extrapolated over the previous 15 min period to determine total grams of exogenous carbohydrate oxidised in each time period from 60 min of exercise onwards. Only the second hour of exercise was considered as exogenous carbohydrate oxidation rates are stable. The total exogenous carbohydrate oxidised was subtracted from the total carbohydrate oxidised over the same time period to give an estimate of endogenous carbohydrate oxidation. The endogenous and exogenous carbohydrate oxidised totals were then multiplied by 4.07 to provide total carbohydrate energy expenditure in kcal for each carbohydrate source. The total fat oxidised was multiplied by 9.75 to give total energy expenditure (kcal) for fat oxidation [13]. The total energy expenditure from all three substrates was then summed and each component was expressed as a percentage of the total energy expenditure over the second hour of exercise.

#### 2.8.6. Glucose Kinetics

From the 6,6,^2^H_2_ tracer infusion the Ra and Rd of glucose were calculated with the single pool non-steady state equations of Steele, as modified for use with stable isotopes [14]. Total Ra represents the total splanchnic glucose from ingested CHO and liver derived glucose:

R_a_ total = F − (pV × (C_1_ + C_2_)/2 × (E_2_ − E_1_)/(*t*_2_ − *t*_1_))/(E_2_ + E_1_)/2) (5)

R_d_ total = R_a_ total − V × (C_1_ + C_2_/*t*_2_ − *t*_1_) (6)

where F is the infusion rate (mg·kg^−1^·min^−1^); E_l_ and E_2_ are the [^2^H_2_] glucose enrichments (MPE) in plasma at time points *t*_1_ and *t*_2_ (min), respectively; C_1_ and C_2_ are glucose concentrations (mg·mL^−1^) at *t*_1_ and *t*_2_, respectively; and pV is volume of distribution which was set at 40 mL·kg^−1^ to coincide with the findings of Wolfe et al. [15].

#### 2.8.7. Estimation of Liver Glucose Contribution

Liver glucose contribution has been estimated from the following calculation:

Whole body glucose Ra (Ra_body_) g·min^−1^ = Ra × body mass × 1000 (7)

Estimation of liver glucose contribution to glucose Ra (%) = 100 − ((EXO/Ra_body_) × 100) (8)

where Ra is the total Ra previously calculated (mg·kg^−1^·min^−1^), and the body mass is the pre-trial body mass measure taken before each trial (kg). The factor of 1000 is to convert from mg to grams. EXO is the exogenous CHO oxidation rate (g·min^−1^) calculated previously. These calculations serve as an estimation of hepatic glucose contribution during the second hour of exercise [5].

### 2.9. Data Presentation and Statistical Analysis

All data are presented as mean (±SD) unless otherwise stated. Unfortunately, two participants had to be removed from all stable isotope and substrate use data due to measurement errors making these data *n* = 18. Specific reference to how many participants are included in each dataset is made for each variable considered. Three factor repeated measures analysis of variance was used to determine treatment, time, period (order) main effects and treatment x time interaction effects. Where a significant period effect was observed then period was used as a covariate and the analysis re-run. Significant main and interaction effects were explored using post hoc Tukey’s comparisons to indicate where these differences occurred. Pearson correlation analysis was performed to examine associations between individual metabolic parameters and the performance outcome differences on 20, 39 and 64 g·h^−1^ trials compared to 0 g·h^−1^. Stepwise linear regression analysis was used to find the best prediction model for performance outcome using multiple metabolic parameters. An alpha value of 0.15 was used for inclusion and exclusion of variables from the model at any given step. An alpha value of 0.15 was chosen to ensure variables were not included or excluded too easily from the model. In addition, a best subsets regression analysis was performed on all metabolic variables. In all cases statistical significance was accepted at *p* < 0.05.

## 3. Results

### 3.1. Participants

Twenty male competitive cyclists completed all trials in this study. All treatments were tolerated well by participants. Tremendous effort was made to ensure all data points were collected, though some data sets had to be removed due to technical problems. As such, all data for substrate oxidation are for *n* = 18 due to the absence of ^13^C tracer on one CHO trial for one participant, and expired gas analysis analytical problems during exercise with one other participant. All other data are for *n* = 20.

### 3.2. Performance Task Outcomes

Performance task data (*n* = 20) has been reported elsewhere [7]. Briefly, endurance cycling performance was equally improved with carbohydrate provision when ingested at a rate of 39 or 64 g·h^−1^ in comparison to water placebo (0 g·h^−1^). No significant difference in performance task time was noted between 20 g·h^−1^ and 0 g h^−1^ treatments, or between 39 and 64 g·h^−1^ treatments (Table 1).

### 3.3. Substrate Oxidation

#### 3.3.1. Respiratory Exchange Ratio

**Figure 2:**
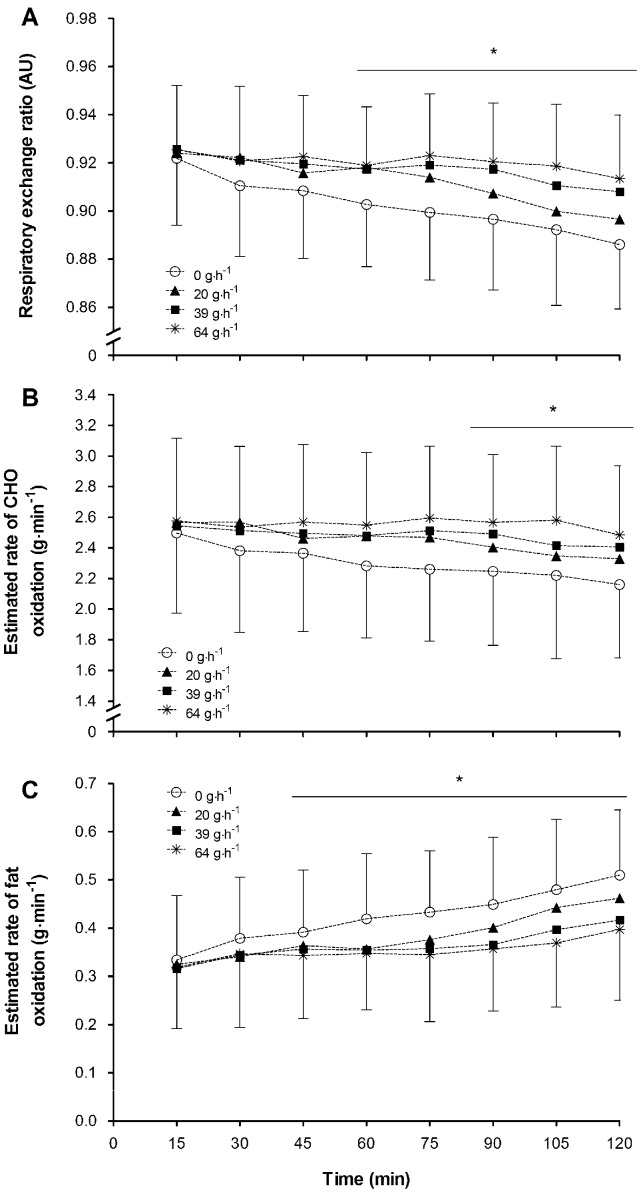
Mean (SD) (**A**) respiratory exchange ratio; (**B**) estimated rate of carbohydrate oxidation and (**C**) estimated rate of fat oxidation during submaximal exercise when consuming 0, 20, 39 and 64 g·h^−1^ of CHO. RER values are significantly (* *p* < 0.01) lower from time point 60 min onwards in comparison to 15 min values, with the 0 g·h^−1^ treatment eliciting a significantly (*p* < 0.01) lower mean RER over the two hours in comparison to 20, 39 and 64 g·h^−1^. A comparison of time indicated CHO oxidation rates at 90 min onwards were significantly lower than 15 min with the 0 g·h^−1^ treatment being significantly lower than 20, 39 and 64 g·h^−1^. Post hoc comparisons indicated the mean rate of fat oxidation was significantly lower when consuming 39 and 64 g·h^−1^ of CHO compared to 0 g·h^−1^. Additionally, time comparisons indicated an increase in fat oxidation from 45 min onwards in comparison to rates at 15 min.

RER data analysis (*n* = 18) revealed a significant main effect of time (*p* < 0.01), treatment (*p* < 0.01), and period (*p* < 0.01) but no interaction (*p* = 0.39). Period was subsequently treated as a covariate for all further analyses. Pairwise comparisons of time indicated that RER values declined with exercise duration. Comparisons of treatment indicated the 0 g·h^−1^ treatment RER was significantly (*p* < 0.01) lower in comparison to 20, 39 and 64 g·h^−1^ (Figure 2A). Mean (SD) RER on the trials was 0.90 (0.03), 0.91 (0.03), 0.92 (0.03) and 0.92 (0.03) for 0, 20, 39 and 64 g·h^−1^ respectively.

#### 3.3.2. Whole Body Substrate Oxidation

Analysis of the carbohydrate oxidation data (*n* = 18) indicated a significant effect of time (*p* < 0.01), treatment (*p* < 0.01), and period (*p* < 0.01) but no interaction effect (*p* < 0.58). Period was treated as a covariate for all subsequent analysis. Pairwise comparisons over time indicated that estimated rate of CHO oxidation was declining over time with measures from 90 min onwards being significantly lower than 15 min values. Treatment pairwise comparisons revealed that the lowest CHO oxidation rate occurred when consuming the 0 g·h^−1^ treatment with significant increases in oxidation when consuming 20 and further increases when consuming 39 and 64 g·h^−1^ in comparison to 20 g·h^−1^ (Figure 2B). Total CHO oxidation on the trials was 279 (58), 298 (52), 302 (47) and 312 (56) g for 0, 20, 39 and 64 g·h^−1^ respectively of which 0 was significantly different (*p* < 0.01) from 64 g·h^−1^.

Results for estimated rate of fat oxidation (*n* = 18) indicated a significant effect of time, treatment and period (all *p* < 0.01) but no interaction effect (*p* = 0.82). Period was treated as a covariate with all further comparisons. Pairwise comparisons of time indicated an increase in fat oxidation rates with increase in exercise duration. Fat oxidation was higher from 45 min onwards compared with 15 min values. Additional pairwise comparisons revealed that consuming the 0 g·h^−1^ treatment resulted in the significantly higher mean fat oxidation rates in comparison to 39 and 64 g·h^−1^ (Figure 2C). Estimated total fat oxidation was 51 (15), 45 (15), 43 (15) and 42 (15) g for 0, 20, 39 and 64 g·h^−1^ respectively of which no total oxidation was significantly different from another.

#### 3.3.3. Exogenous Carbohydrate Oxidation and Whole-Body Substrate Contribution to Total Energy Expenditure

**Figure 3:**
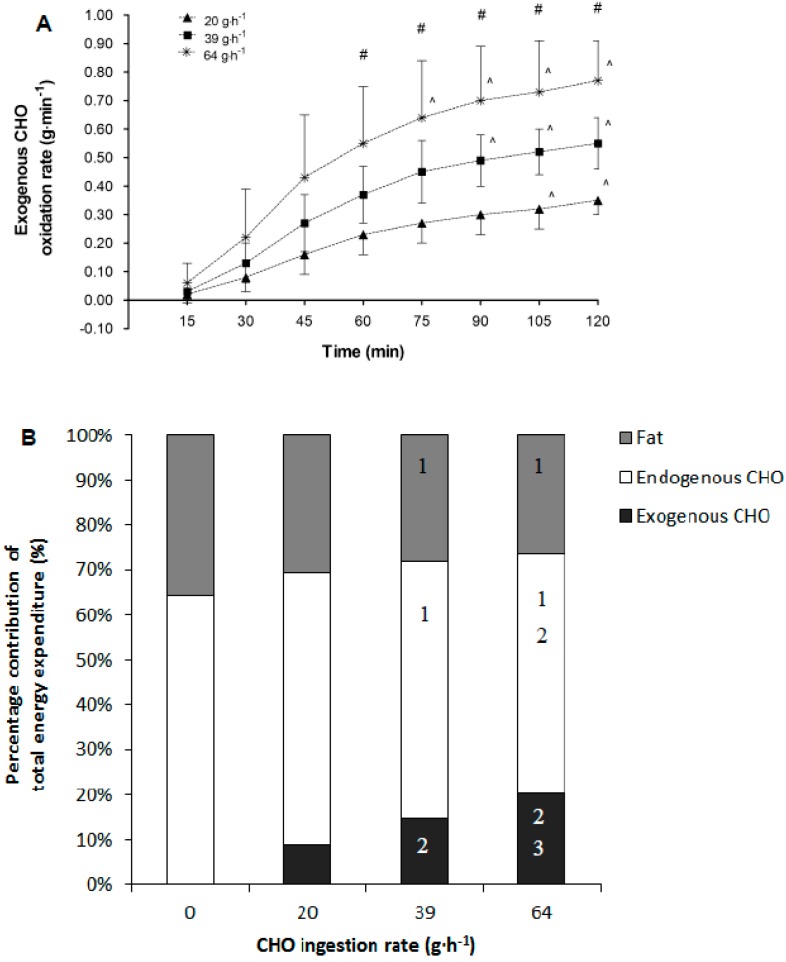
Mean (SD) exogenous carbohydrate (CHO) oxidation rates during submaximal exercise (**A**) while consuming 0, 20, 39 and 64 g·h^−1^ of CHO. ^ indicates time point values significantly (*p* < 0.01) different in comparison to 60 min values; # indicates that all trials are significantly (*p* < 0.01) different from each other at the indicated time point; (**B**) Percentage contribution of total carbohydrate oxidation rates from endogenous and exogenous sources during the second hour of exercise; 1 Indicates significantly different from 0 g·h^−1^; 2 indicates significantly different from 20 g·h^−1^; and 3 indicates significantly different from 39 g·h^−1^.

Data for exogenous carbohydrate oxidation (*n* = 18) indicated significant main effects of treatment (*p* < 0.01), time (*p* < 0.01), period (*p* < 0.01) and an interaction effect between treatment and time (*p* < 0.01). Period was included as a covariate for all further comparisons. Pairwise comparisons indicated that exogenous CHO oxidation rates were significantly different between all treatments from the 60-min time point until the end of exercise. Specifically, exogenous CHO oxidation rates were higher in comparison to the 20 g·h^−1^ treatment by 0.13 (95% CI: 0.10 to 0.15) and 0.29 (95% CI: 0.27–0.31) g·min^−1^ on the 39 and 64 g·h^−1^ treatments. Additionally, the 64 g·h^−1^ treatment increased exogenous oxidation rates at 75, 90, 105 and 120 min above the 60 min rates highlighting that exogenous CHO oxidation was still rising from 60 min onwards on this trial. Similarly, when consuming 39 g·h^−1^ the values at 90, 105 and 120 min also were significantly increased above the 60 min values, and at 105 and 120 min in comparison to 60 min for the 20 g·h^−1^ treatment (Figure 3A).

Percentage contribution of fat oxidised in the second hour of exercise (*n* = 18) revealed significant effects of treatment (*p* < 0.01) and period (*p* < 0.01). Following inclusion of period as a covariate, pairwise comparisons of treatment indicated the percentage contribution of fat oxidation was significantly lower when consuming 39 (−7.5, 95% CI: −1.6 to −13.4%) and 64 (−8.9, 95% CI: −3.1 to −14.8%) g·h^−1^ in comparisons to consuming 0 g·h^−1^. Endogenous carbohydrate percentage contribution highlighted significant effects of treatment (*p* < 0.01) but not period (*p* = 0.39). Pairwise comparisons of treatment indicated that endogenous carbohydrate percentage contribution was significantly suppressed in the 39 and 64 g·h^−1^ trials (−7.3, 95% CI: −1.6 to −13.1 and −11.2, 95% CI: −5.5 to −16.9 respectively) compared to the 0 g·h^−1^ treatment, respectively. Additionally, consuming carbohydrate at 64 g·h^−1^ suppressed endogenous carbohydrate percentage contribution by −7.2% (95% CI: −1.5–13.0%) in comparison to the 20 g·h^−1^ treatment. Exogenous carbohydrate oxidation percentage contribution demonstrated a significant effect of treatment (*p* < 0.01). Pairwise comparisons indicated that for exogenous carbohydrate oxidation all treatments were significantly different from one another (Figure 3B).

**Figure 4:**
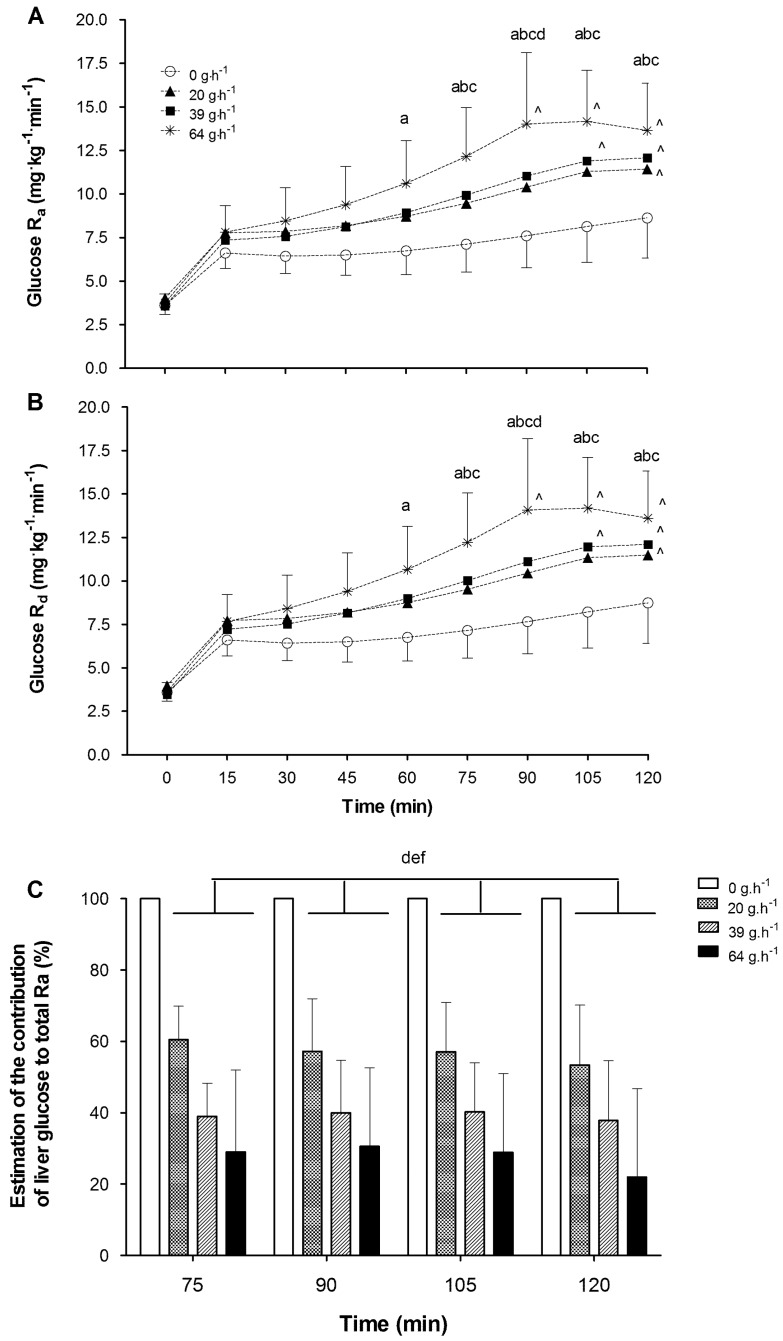
Mean (SD) glucose rate of appearance (**A**) and rate of disappearance (**B**) during submaximal exercise while consuming 0, 20, 39 and 64 g·h^−1^ of CHO. a, b and c indicates 64, 39 and 20 g·h^−1^ value is significantly different from 0 g·h^−1^ at the marked time point; d indicates 64 g·h^−1^ is significantly different from 20 g·h^−1^ at marked time point. ^ indicates time point values significantly (*p* < 0.01) different in comparison to 60 min values. Mean (SD) estimation of the contribution of liver glucose to total glucose Ra during submaximal exercise (**C**) while consuming 20, 39 and 64 g·h^−1^ of CHO. There was a significant treatment effect whereby 64 g·h^−1^ was significantly different from 20 g·h^−1^ (d) and 39 g·h^−1^ (e); and 39 g·h^−1^ was significantly (*p* < 0.01) different from 20 g·h^−1^ (f).

Glucose Ra values (*n* = 18) were mirrored by that of the Rd values, and as such statistical analysis outcomes for both data sets were almost identical. Analysis of the glucose Ra and Rd indicated significant effects of treatment (*p* < 0.01), time (*p* < 0.01), period (*p* < 0.01) and an interaction of treatment by time (*p* < 0.01). Period was treated as a covariate for all subsequent analysis. Post hoc comparisons revealed that consuming CHO resulted in a significantly higher glucose Ra of 1.98 (95% CI: 1.37–2.58), 2.12 (95% CI: 1.52–2.72), and 3.65 (95% CI: 3.05–4.25) mg·kg^−1^·min^−1^ for the 20, 39 and 64 g·h^−1^ treatments respectively, when compared to the 0 g·h^−1^ condition. Post hoc interaction comparisons indicated a significant increase in glucose Ra when consuming 39 and 64 g·h^−1^ compared to 0 g·h^−1^ at time points from 75 min onwards. Additionally, during the 20 g·h^−1^ trial glucose Ra was significantly increased over the control condition at time points from 90 min onwards. Glucose Ra values were significantly increased over the 60 min time point value from 75 to 120 min in the 64 g·h^−1^, 105 to 120 min with 39 g·h^−1^, and only at 120 min in the 20 g·h^−1^ Trial (Figure 4A,B).

In the analysis of the contribution of liver glucose to total Ra (*n* = 18), significant effects of treatment (*p* < 0.01) but not time (*p* = 0.13) or interaction (*p* = 0.89) were observed. Pairwise comparisons of treatment indicated that the percentage contribution of liver glucose to total Ra was significantly reduced between 20 and 39 g·h^−1^ feeding rates (−17.8%, 95% CI: −22.8 to −12.8%), and further significantly reduced when comparing 39 to 64 g·h^−1^ (−11.6%, 95% CI: −16.6 to −6.6%). These reductions represent a mean percentage suppression of liver glucose output of 43 (14%), 61 (14%) and 72 (23%) for the 20, 39 and 64 g·h^−1^ treatments, respectively, in comparison to the 0 g·h^−1^ (Figure 4C).

### 3.4. Blood Plasma Measures

#### 3.4.1. Glucose

**Figure 5:**
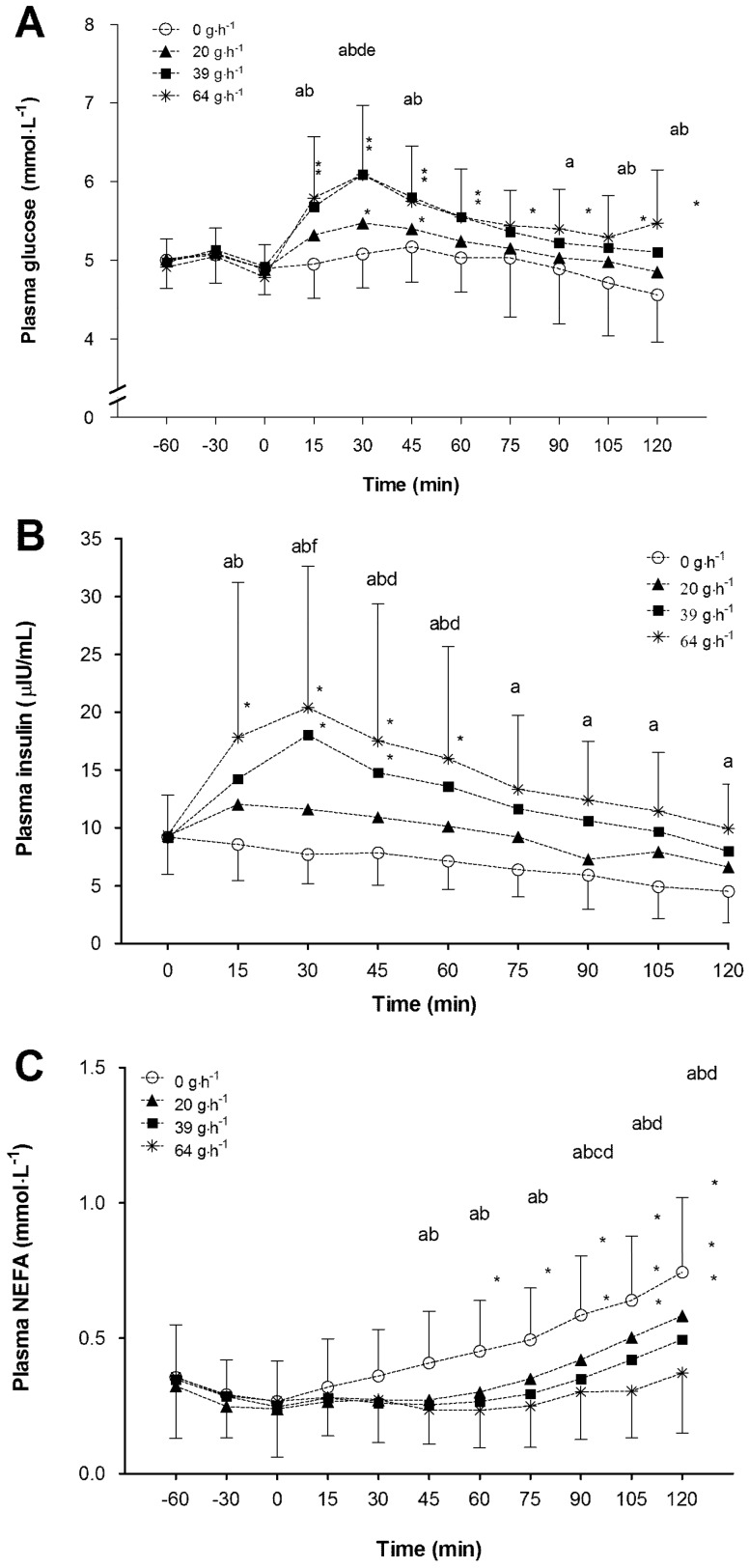
Mean (SD) plasma glucose (**A**); insulin (**B**); and non-esterified fatty acids (**C**) concentration during rest (−60, −30 and 0 min), and during submaximal exercise, (15–120 min), while consuming 0, 20, 39 and 64 g·h^−1^ of CHO. * Values significantly different from 0 min time point. a, b and c indicates 64, 39 and 20 g·h^−1^ value is significantly different from 0 g·h^−1^ at the marked time point; d indicates 64 g·h^−1^ is significantly different from 20 g·h^−1^ at marked time point; e indicates 39 g·h^−1^ is significantly different from 20 g·h^−1^ at marked time point; f indicates 39 and 64 g·h^−1^ are both different from the 20 g·h^−1^ at the marked time point.

There were main effects of time (*p* < 0.01), treatment (*p* < 0.01), period (*p* < 0.01) and an interaction effect (*p* < 0.01) of treatment by time observed for plasma glucose response (*n* = 20). Period was then used as a covariate for all further analyses. Mean glucose concentration was higher when consuming 39 g·h^−1^ and 64 g·h^−1^ (0.41 mmol·L^−1^ (95% CI: 0.31–0.51) and 0.46 mmol·L^−1^ (95% CI 0.36–0.56), respectively) when pairwise comparisons to the 0 g·h^−1^ treatment were made. Consuming 39 and 64 g·h^−1^ also resulted in increased mean plasma glucose concentration by 0.23 (95% CI: 0.13–0.33) and 0.28 (95% CI: 0.18–0.38) mmol·L^−1^, respectively, in comparison to consuming 20 g·h^−1^. There was no evidence of a difference between 39 and 64 g·h^−1^ treatments. Treatment by time interaction analysis revealed that plasma glucose concentration was significantly increased above 0 min in the 64 g·h^−1^ treatment from 15 min until the end of the exercise period. Additionally, the 39 g·h^−1^ treatment significantly increased plasma glucose concentration from the 0 min value at 15, 30, 45 and 60 min, as did the 20 g·h^−1^ treatment at 30 and 45 min (Figure 5).

#### 3.4.2. Insulin

There were main effects of time (*p* < 0.01), treatment (*p* < 0.01), and an interaction effect between treatment and time (*p* < 0.01) on plasma insulin response (*n* = 20). There was no effect of period (*p* = 0.14). On average insulin concentration increased by 2.5 (95% CI: 1.3–3.7), 5.2 (95% CI: 4.0–6.4) and 7.3 (95% CI: 6.1–8.5) µIU·mL when consuming 20, 39 and 64 g·h^−1^ CHO, respectively, compared to the 0 g·h^−1^ trial. Insulin concentration significantly increased from pre ingestion (0 min) values at 15–60 min time points for 64 g·h^−1^, and at 30 and 45 min for 39 g·h^−1^. Further pairwise comparisons revealed that insulin concentration was significantly elevated in the 39 and 64 g·h^−1^ treatments when compared to the 0 g·h^−1^ at time points between 15 and 45 min. At 30 min, consuming 39 and 64 g·h^−1^ also significantly elevated insulin concentration over that of consuming 20 g·h^−1^. The 64 g·h^−1^ treatment also significantly increased insulin concentration at time points 45 and 60 min when compared to the 20 g·h^−1^ treatment (Figure 5).

#### 3.4.3. Non-Esterified Fatty Acids

There were main effects of time (*p* < 0.01), treatment (*p* < 0.01), period (*p* < 0.01) and an interaction of treatment by time (*p* < 0.01) on plasma NEFA (*n* = 20). Period was included as a covariate for all further analyses. Pairwise comparisons between treatments revealed that on the 0 g·h^−1^ treatment mean NEFA concentration was 0.10 (95% CI: 0.07–0.13), 0.12 (95% CI: 0.10–0.16) and 0.16 (95% CI: 0.13–0.19) mmol·L^−1^ higher than when consuming the 20, 39 and 64 g·h^−1^ treatments, respectively. Additionally, the NEFA concentration throughout exercise on 20 g·h^−1^ was significantly higher (0.06 mmol·L^−1^, 95% CI: 0.03–0.09) than when consuming 64 g·h^−1^. When consuming 0 g·h^−1^ all NEFA concentrations were significantly increased above the 0 min time point from 60 min onwards. On the 20 g·h^−1^ treatment plasma NEFA concentration was elevated compared to the 0 min time point at 90, 105 and 120 min. Additionally, on the 39 g·h^−1^ treatment NEFA concentration increased at time points 105 and 120 min compared to 0 min. No increase was observed on 64 g·h^−1^ treatment. Post hoc interaction comparisons revealed that mean NEFA concentration in the 0 g·h^−1^ treatment was significantly elevated compared to the 39 and 64 g·h^−1^ treatments from the 45 min time point until the end of exercise. Additionally, the 20 g·h^−1^ treatment was significantly different from 64 g·h^−1^ at 90, 105 and 120 min. Finally, the 20 g·h^−1^ treatment significantly elevated plasma NEFA concentration in comparison to the 0 g·h^−1^ at time point 90 min (Figure 5).

#### 3.4.4. Lactate

Plasma lactate concentration (*n* = 20) revealed a significant effect of time (*p* < 0.01) and an effect of treatment (*p* = 0.02) but no interaction (*p* = 0.84), and no period effect (*p* = 0.57). Post hoc comparisons of time indicated that all exercising lactate concentrations were elevated above resting values, though there was no significant difference between trials. Mean plasma lactate concentration was 1.06 (0.38), 1.09 (0.35), 1.04 (0.29) and 1.10 (0.36) mmol·L^−1^ for 0, 20, 39 and 64 g·h^−1^ trials, respectively.

#### 3.4.5. Adrenaline

Analysis of adrenaline concentration (*n* = 20) revealed there was a main effect of period (*p* < 0.01) time (*p* < 0.01), treatment (*p* < 0.01), but no interaction (*p* = 0.10). Period was treated as a covariate for all subsequent analysis. Pairwise comparisons of time indicated adrenaline concentrations were increasing over the duration of the exercise bout. Additionally, comparisons between treatments indicated that adrenaline concentration was highest on the 0 g·h^−1^ trial (0.99 ± 0.69 ng mL^−1^) in comparison to the 39 g·h^−1^ (0.78 ± 0.38 ng mL^−1^) and 64 g·h^−1^ (0.78 ± 0.41 ng mL^−1^) trials. Mean adrenaline concentration on the 20 g·h^−1^ trial was 0.87 (0.48) ng mL^−1^.

### 3.5. Associations between Metabolic Responses and Prediction of Subsequent Performance Outcomes

Association analysis between a number of key metabolic parameters during 2 h of exercise and subsequent change in performance task outcome are shown in Table 2. This analysis revealed a moderate positive association between increases in total exogenous CHO oxidized in the second hour of exercise, and total glucose rate of disappearance in the second hour of exercise, with an improvement in performance outcome. In addition, there was a tendency towards a moderate negative association between change in circulating NEFA concentration and subsequent performance outcome. Factors such as liver glucose output suppression, mean plasma glucose, and mean plasma insulin concentrations were not associated with the changes in performance task outcome.

Using these metabolic parameters in a stepwise linear regression analysis revealed that the combination of exogenous CHO oxidation in the second hour of exercise (*p* = 0.011) and the suppression of circulating NEFA (*p* = 0.010) provided the best model to predict subsequent performance outcomes as shown in Table 3. However, this model only explained ~19% of the variance in performance outcome observed. The regression equation that resulted was:

Performance change from 0 (%) = 1.13 + 0.405 (Exo CHO) − 34.6 (Δ NEFA)(9)

where Exo CHO is the total exogenous CHO oxidation in 2nd hour in grams and Δ NEFA is the difference in NEFA concentration (mmol·L^−1^) in comparison to consuming the 0 g·h^−1^. Finally, a best subsets analysis was conducted on all variables included in the regression. Including all variables reported in Table 2, except for mean glucose concentration, explained 23% of the variance in performance.

### 3.6. Heart Rate and RPE

There were significant effects of time, treatment and period (*p* < 0.01) but no interaction effect (*p* > 0.99) for heart rate. Period was treated as a covariate for all subsequent analysis. Pairwise comparisons indicated that heart rate tended to increase with increasing exercise duration. In addition, post hoc comparisons of treatment indicated a significant difference between 0 and 64 g·h^−1^ with a mean difference of 4 (95% CI: 2, 6) beats per minute between the two trials. The mean heart rates for each treatment were 135, 137, 136 and 139 for 0, 20, 39 and 64 g·h^−1^ respectively.

There was a significant effect of time (*p* < 0.01) and period (*p* = 0.02), but not treatment (*p* = 0.83) or interaction (*p* = 0.94) effects on RPE responses to exercise. Period was treated as a covariate for all further comparisons. Post hoc comparisons indicated that mean RPE scores increased from 13 to 14 from minute 60 to minute 120.

## 4. Discussion

During this investigation, we aimed primarily to characterize the metabolic response of trained cyclists to the ingestion of graded amounts of CHO during a two-hour submaximal ride to explore the dose-response. Our secondary aim was to determine the strength of association between selected metabolic parameters and prediction of the performance task outcomes, previously reported elsewhere [7]. We observed that increasing rates of CHO ingestion (particularly at 39 and 64 g·h^−1^) during non-exhaustive submaximal exercise resulted in: (1) a reduction in the contribution of endogenous carbohydrate and fat stores to total energy provision; (2) a decrease in hepatic glucose output in a dose response manner; (3) an increase in the contribution of exogenous CHO oxidation to total energy contribution in a dose response manner; (4) an increase in rate of total carbohydrate oxidation and plasma glucose turnover; (5) increased circulating blood glucose and insulin concentration; and (6) a blunting of the circulating NEFA response to exercise. While individually these responses to increasing doses of CHO feeding are not unforeseen the novel aspect of the present study is the examination of dose-response effects in all of these responses. Moreover, the correlation and regression analyses indicate that the rate of exogenous CHO oxidation and suppression of NEFA are the two most closely linked to a significant prediction of subsequent performance task outcome.

The significant alterations in fuel selection observed with the ingestion of 39 and 64 g·h^−1^ of CHO in comparison to 0 or 20 g·h^−1^ closely compliment the performance task outcome data previously reported [7]. In addition, the ingestion of 64 g·h^−1^ had no added effect, over 39 g·h^−1^, on many of the key metabolic responses to exercise, but it did result in an increased rate of exogenous substrate oxidation, and a further blunting of hepatic glucose output, when compared with the 39 g·h^−1^ trial. A difference in exogenous CHO oxidation rate and hepatic glucose output, two key metabolic parameters, between the 39 and 64 g·h^−1^ trials might be expected to impact subsequent performance, but no impact was observed. Interestingly, the best subsets regression analysis indicated that some other metabolic parameters were also associated with change in performance in relation to graded doses of CHO ingestion. These other predictors were mean insulin concentration, and suppression in circulating plasma NEFA. The lack of difference in insulin or NEFA response between the 39 and 64 g·h^−1^ trials suggests that, with ingestion of a dose of single source CHO up to 64 g·h^−1^ over a two-hour exercise bout, there seems little metabolic advantage of going beyond ~40 g·h^−1^.

Many investigators have observed a significant difference in plasma insulin and NEFA concentration with the ingestion of CHO during submaximal exercise, and a subsequent alteration of fuel utilization [5,16]. However, the only previous dose-response study [4] did not observe differences in insulin or NEFA response between the two highest CHO doses ingested (30 and 60 g·h^−1^). The present study data corroborate these observations and, with a more suitably powered design, suggest that moderate amounts of CHO in the region of only 40 g·h^−1^ are sufficient to modulate metabolic responses enough to impact upon subsequent performance task outcome. By utilizing stable isotopes researchers have been able to quantify the movement of glucose into and out of the plasma pool during exercise when carbohydrate is consumed [15]. During exercise, blood glucose can be maintained or increased by the augmented release of glucose from the liver. In the present study, the 20 g·h^−1^ treatment reduced hepatic glucose output by 43% but the performance outcome (3.7% improvement) previously reported [7] was too variable for it to be considered a significant performance enhancement. Hepatic glucose output calculations in the current investigation reveal that all CHO ingestion rates resulted in a reduction in hepatic glucose output, and that the magnitude of reduction essentially followed a dose-response pattern. Higher CHO ingestion rates of 39 and 64 g·h^−1^ both suppressed hepatic glucose output to a greater extent than the 20 g·h^−1^ treatment. Interestingly, the magnitude of suppression in the 39 and 64 g·h^−1^ trials was similar or greater than that observed by McConnell et al. [8] when they fed CHO at 100 g·h^−1^. This observation could suggest that even low doses of CHO at 39 and 64 g·h^−1^ are resulting in a near maximal suppression of hepatic glucose output, which is not exceeded unless very high doses of CHO are ingested (i.e., 180 g·h^−1^; Jeukendrup [5]). The lack of any association between hepatic glucose output suppression and performance outcome suggests that even modest reductions in liver glucose output, induced by feeding only 39 g·h^−1^ of CHO during two hours of exercise, are sufficient to impact upon subsequent endurance task performance.

Exogenous CHO oxidation rates increase when CHO is ingested, but when a single source of CHO is ingested these typically only reach rates of ~1 g·min^−1^ [5,17,18]. In the present study, rates of exogenous CHO oxidation followed a dose-response pattern with the highest rates of around 0.75 g·min^−1^ achieved on the 64 g·h^−1^ trial. On the 39 g·h^−1^ trial exogenous CHO oxidation rates reached 0.55 g·min^−1^. These data are slightly higher than those obtained by Smith et al. [4] in their dose-response study. Smith et al. [4] noted rates of ~0.3 and ~0.5 g·min^−1^ for their 30 and 60 g·h^−1^ CHO ingestion trials, respectively. The lack of performance task improvement with increasing rate of oxidation of exogenous CHO in the present study, and in the Smith et al. [4] study, suggests that capacity to oxidize exogenous CHO at a high rate is not for the key factor driving performance improvement. In addition, there was only a weak, albeit significant, association observed between exogenous CHO oxidation rate and subsequent performance outcome, as well as a modest contribution from exogenous CHO oxidation rate to prediction of performance outcome in the regression analyses. Overall, these data suggest that higher exogenous CHO delivery and higher subsequent oxidation likely contribute to endogenous (hepatic) glycogen sparing during two hours of endurance cycling, and can have some impact upon subsequent performance task outcome. However, as a note of caution, these observations may be particular to the exercise model investigated. For example, in longer exercise task durations exceeding three hours of total activity it may well is that higher feeding rates and higher exogenous CHO oxidation would translate to improved performance.

The blunting of fat oxidation observed only on the two highest CHO doses (39 and 64 g·h^−1^) subsequently would drive fuel utilization towards a CHO dependent state. The suppression of fat oxidation and circulating NEFA concentration was similar on both 39 and 64 g·h^−1^ feeding rates. Thus, it seems that feeding of only 39 g·h^−1^ is sufficient to sustain exercise. Van Loon et al. [19] reported that a suppression in adipose tissue lipolysis increases glycogen utilization in exercising humans. While a greater dependence upon CHO oxidation was observed between 39 and 64 g·h^−1^ feeding rates compared with 0 g·h^−1^, there was no difference in CHO usage between the two highest feeding rates in the present study. These observations suggest that near optimal substrate metabolism changes occurred with a feeding rate of close to 40 g·h^−1^.

Our results highlight that CHO provision leading to an increased oxidation of exogenous CHO, increased total glucose disposal, and reduction in circulating NEFA, have the closest associations with subsequent performance task outcome. Higher CHO feeding rates that reach a threshold level to blunt circulating NEFA concentration, increase reliance on CHO oxidation, and enhance exogenous CHO oxidation, will have the biggest impact upon subsequent performance task outcome. The threshold required for these outcomes appears to be around 40 g·h^−1^ in the present study. However, these associations are low to moderate and the threshold of CHO ingestion rate could well be influenced by the total task duration and/or training status of participants. With longer task durations (>3 h) an increased reliance on exogenous CHO oxidation later in exercise could enhance liver glycogen sparing and could improve subsequent performance outcomes. Furthermore, with improved training status comes improved capacity to oxidize substrates [20,21] which might drive the CHO provision threshold beyond 40–60 g·h^−1^.

Prediction of performance task outcome from metabolic parameters was not particularly strong, with only 19–23% of the variance in subsequent performance task outcome explained by the key metabolic parameters in the model. Interestingly, the prediction model containing only the two variables of exogenous CHO oxidation rate and suppression of plasma NEFA response provided almost all of the predictive power of the model. Given that these two parameters are most closely aligned to the actual CHO dose administered, it would seem plausible to suggest that higher doses of CHO should result in better performance outcomes. However, further investigation into higher rates of CHO provision and performance outcome are required before this can be categorically stated. Of particular interest would be studies in which comparisons are made between ingestion rates within the 40–60 g·h^−1^ range using single source CHO, with those in the 90–120 g·h^−1^ range using multiple transportable CHO. To date, only one study, by Baur et al. [22], has compared a single source trial with a practically relevant feeding rate of glucose, against a multiple transportable CHO trial designed to maximize rate of exogenous CHO oxidation. Their study compared feeding rates of glucose at 62 and 93 g·h^−1^ with a 2:1 glucose: fructose beverage ingested at 93 g·h^−1^, during three hours of endurance cycling. Their data revealed that when compared to the 62 g·h^−1^ glucose trial, there was no clear evidence of a benefit to performance compared with ingestion of 93 g·h^−1^ of the glucose: fructose beverage. These data indicate that aiming to increase exogenous CHO oxidation through consumption of composite CHO drinks at high feeding rates will not necessarily lead to meaningful performance gains. Thus, it seems that there is a need for further investigation of CHO dose and performance outcome. So, at high ingestion rates the use of multiple transportable CHO will likely reduce gastrointestinal symptoms, but this does not necessarily translate into enhanced endurance exercise performance. These previous findings may explain the lack of a strong association between exogenous CHO oxidation rate and subsequent performance outcome in the present dataset. Thus, it seems that in endurance tasks lasting <3 h, a feeding rate of 40 g·h^−1^ of single source CHO could be considered near optimal to provide sufficient metabolic advantages to maximize performance gains.

## 5. Conclusions

Researchers have been aiming to identify the optimal ingestion rate of CHO to elicit the greatest improvements in endurance performance for many years. We have reported that the ingestion of 39 and 64 g·h^−1^ of single source CHO were equally effective at improving endurance exercise performance in comparison to a control condition (0 g·h^−1^). The data presented in the current manuscript further demonstrate that the ingestion of 39 g·h^−1^ of CHO appears sufficient to alter substrate utilization during a two-hour submaximal exercise bout, and lead to performance gains. These performance gains partly come from preservation of endogenous glycogen stores, most likely hepatic stores, and maintaining high rates of CHO oxidation through suppression of circulating NEFA concentration. Ingestion of CHO at a lower rate (20 g·h^−1^) is insufficient for these particular metabolic changes to occur, while increasing the rate of ingestion to 64 g·h^−1^ does not appear to have any additional benefit. The lack of any additional change in many metabolic parameters when consuming 64 g·h^−1^ could be partly responsible for a lack of any additional improvement in subsequent performance task outcome. From these present observations, we conclude that an ingestion rate of 39 g·h^−1^ is a dose beyond which there a no further performance or metabolic benefits, suggesting that it could be an optimal ingestion rate, to elicit a sufficient alteration in fuel provision during submaximal exercise. While a 39 g·h^−1^ dose appears effective in this investigation, the observations should be confined to the particular task duration and participant group studied. Further work is required to explore the metabolic advantages and potential performance enhancement from higher feeding rates in more well-trained/elite competitors, in female participants, and in tasks lasting longer than three hours.

## Figures and Tables

**Table 1:** Performance task outcome data on trials where 0, 20, 39 and 64 g·h^−1^ of carbohydrate was ingested.

Variable	Performance Time (min)	Percentage Change from 0 g·h^−1^ (%)	Cohen’s Size Effect from 0 g·h^−1^
0 g·h^−1^	37:01.9 ± 05:35.0	-	-
20 g·h^−1^	35:17.6 ± 04:16.3	3.7 (95% CI −1.5–8.8; *p* = 0.13)	0.6 (95% CI −0.1–1.4)
39 g·h^−1^	34:19.5 ± 03:07.1	6.1 (95% CI 1–11.3; *p* = 0.02)	1.0 (95% CI 0.2–1.7)
64 g·h^−1^	34:11.3 ± 03:08.5	7.0 (95% CI 1–12, *p* = 0.01)	1.0 (95% CI 0.3–1.8)

**Table 2:** Association analysis for selected metabolic parameters, obtained on the 20, 39 and 64 g·h^−1^ ingestion trials, with change in performance outcome compared to 0 g·h^−1^.

Variable	Pearson’s Correlation	*p*-Value
Liver glucose suppression (%)	−0.055	0.691
Total exogenous CHO oxidation in 2nd hour (g)	0.269	0.049 *
Total glucose Rd in 2nd hour (g)	0.291	0.033 *
Mean glucose concentration	−0.045	0.747
Mean insulin concentration	0.158	0.253
Δ NEFA concentration from 0 g·h^−1^	−0.262	0.056 ^†^

* Significant association between parameter and change in performance outcome from 0 g·h^−1^ trial; ^†^ tendency towards significant association.

**Table 3:** Stepwise regression analysis of metabolic variables, obtained on the 20, 39 and 64 g·h^−1^ ingestion trials, with change in performance outcome compared to 0 g·h^−1^.

Model	Variable (ID)	*R*^2^	*p* (Variable ID)
1	(1) Total glucose Rd in 2nd hour (g)	0.087	0.033 (1) *
2	(2) +Δ NEFA concentration from 0 g·h^−1^	0.126	0.129 (2), 0.074 (1) *
3	(3) +Total exogenous CHO oxidation in 2nd hour (g)	0.192	0.048 (3) *, 0.032 (2) *, 0.48 (1)
4	(4) −Total glucose Rd in 2nd hour (g)	0.184	0.011 (2) *, 0.010 (3) *

Alpha-to-enter = 0.15; Alpha-to-remove = 0.15; * indicates a significant (*p* < 0.05) component in the model.

## Supplementary Materials

Supplementary File 1
